# Common Myths and Misconceptions About Primary Angioplasty in Acute Myocardial Infarction: An Evidence-Based Perspective for Indian Primary Care Physicians

**DOI:** 10.7759/cureus.95132

**Published:** 2025-10-22

**Authors:** Kunal Mahajan, Jai Bharat Sharma, Surender Himral, Tanuj Bhatia, Ashwani Kumar, Rahul Yadav, Savio Dsouza, Shivali Sandal, Roshan Thakur, Iva Patel

**Affiliations:** 1 Cardiology, Himachal Heart Institute, Mandi, IND; 2 Cardiology, Shri Mahant Indiresh Hospital, Dehradun, IND; 3 Cardiology, Pandit Bhagwat Dayal Sharma Post Graduate Institute of Medical Sciences, Rohtak, IND; 4 Cardiology, Charak Hospital and Research Centre, Lucknow, IND; 5 Cardiology, Kasturba Medical College, Mangalore, IND; 6 Critical Care Medicine, Himachal Heart Institute, Mandi, IND; 7 Internal Medicine, Himachal Heart Institute, Mandi, IND

**Keywords:** india, knowledge gaps, myths and misconceptions, primary pci, st-elevation myocardial infarction (stemi)

## Abstract

Primary percutaneous coronary intervention (PCI) is the gold standard for managing ST-elevation myocardial infarction (STEMI). However, in India, despite the availability of catheterization laboratories, timely access to PCI remains limited due to persistent myths and misconceptions among primary care physicians. Common misconceptions include exaggerated concerns about procedural risks, the belief that medical management or thrombolysis is equally effective, the assumption that patients must be fully stabilized before referral, and uncertainty regarding the role of pharmacoinvasive (PI) strategies. Evidence consistently demonstrates that primary PCI is both safe and highly effective, reducing mortality, reinfarction, and stroke compared with thrombolysis, with very low complication rates. Despite these proven benefits, only a small proportion of STEMI patients in India currently receive primary PCI. Significant delays in care also persist, with many patients reaching the hospital several hours after symptom onset. PI therapy has emerged as a pragmatic alternative when timely PCI cannot be achieved, offering outcomes comparable to primary PCI in resource-limited and geographically constrained settings. Addressing these misconceptions through targeted education, standardized protocols, and strengthened STEMI networks is essential to improve outcomes and optimize reperfusion therapy in India.

## Introduction and background

ST-elevation myocardial infarction (STEMI) is one of the most time-critical emergencies in cardiovascular medicine, where prompt recognition and appropriate treatment directly determine patient outcomes. In India, the burden of acute coronary syndromes is substantial, with an estimated three million STEMI cases occurring annually [1]. Despite strong evidence supporting primary percutaneous coronary intervention (PCI) as the gold standard reperfusion strategy, significant gaps persist in the timely delivery of evidence-based care [2].

The Indian healthcare landscape presents unique challenges in STEMI management. Unlike developed countries, where primary PCI is readily available, India has limited PCI-capable facilities offering round-the-clock services. The CREATE registry (2008), one of India’s largest acute coronary syndrome studies, revealed that only 8% of patients received primary PCI, whereas 59% underwent thrombolysis. Even more concerning is the pattern of delayed presentation, with patients taking a median of 300 minutes (five hours) to reach healthcare facilities, and only 5% arriving via ambulance [3].

Primary care physicians (PCPs) serve as the first point of contact for many STEMI patients in India, particularly in rural and semi-urban areas where specialized cardiac care is limited [4]. However, studies have identified significant knowledge gaps and misconceptions among PCPs regarding current STEMI management guidelines. These myths and misunderstandings contribute to treatment delays, inappropriate referral patterns, and suboptimal outcomes [5]. Common misconceptions include the belief that primary PCI carries a higher procedural risk, is prohibitively expensive, or is unnecessary after thrombolysis, all of which may discourage timely transfer to PCI-capable centers.

In contrast, primary PCI significantly reduces adverse outcomes in STEMI patients and remains the gold standard treatment. Meta-analyses consistently demonstrate that, compared with fibrinolysis, primary PCI lowers short-term mortality, decreases the risk of reinfarction, and substantially reduces stroke rates. These benefits are evident within the first few weeks and persist during long-term follow-up. It remains the most effective reperfusion strategy across patient subgroups and clinical settings, including those requiring transfer, and consistently shows superior safety and efficacy compared with both fibrinolysis and alternative PCI strategies [6,7].

This narrative review aims to address the most prevalent myths about primary angioplasty among Indian PCPs, providing evidence-based clarifications and practical recommendations for improving STEMI care within the Indian healthcare context.

## Review

Prevalent myths among Indian PCPs

Myth 1: Primary Angioplasty Is Excessively Risky

Myth: Many Indian PCPs believe that primary PCI carries unacceptable procedural risks, including high mortality rates and severe complications that outweigh its potential benefits [5].

Evidence-based reality: Contemporary data from multiple registries and clinical studies demonstrate that primary PCI is exceptionally safe and effective. The direct procedural mortality rate is approximately 0.1%, making it one of the safest emergency medical procedures available [8]. Evidence from 23 randomized controlled trials involving over 7,700 patients confirmed that PCI leads to lower short-term mortality (7% vs. 9%), fewer reinfarctions (3% vs. 7%), and a reduced risk of stroke (1% vs. 2%) compared with thrombolysis. These benefits were consistent across all subgroups, regardless of the thrombolytic agent used or the need for patient transfer [7].

Indian registry data further support these findings. The Kerala STEMI registry [9], which included multiple PCI-capable hospitals, reported excellent procedural success rates exceeding 95%, with low complication rates. The safety profile of primary PCI is consistently superior to that of thrombolytic therapy, with markedly lower rates of intracranial hemorrhage (0.05% vs. 0.7%) and major bleeding complications.

STEMI India, a national initiative, addresses urban-rural disparities in STEMI care by implementing a hub-and-spoke model, offering primary PCI in urban centers and a pharmacoinvasive (PI) strategy in rural areas. This approach has been validated in states such as Tamil Nadu and Goa, although challenges such as workforce training and technological limitations persist [9].

Building on these observations, the NORIN registry [10] further highlights persistent gaps in STEMI care across India, revealing substantial delays in reperfusion, underutilization of evidence-based therapies, and significant regional disparities in management, all contributing to higher mortality compared with high-income countries. Consistent with these findings, data from a large North Indian tertiary care registry [11] similarly demonstrate ongoing challenges in STEMI management, including a primary PCI rate of only 18% and considerable delays in presentation, underscoring the urgent need to strengthen referral systems and improve access to timely PCI.

Myth 2: Thrombolysis Is as Effective as Primary PCI for STEMI

Myth: Some Indian PCPs believe that optimal medical therapy, comprising thrombolysis along with antiplatelet agents, anticoagulants, and supportive care, can adequately manage STEMI without the need for invasive intervention [5].

Evidence-based reality: Reperfusion achieved by transferring patients to a PCI-capable center for primary angioplasty is superior to on-site fibrinolysis if the transfer time does not exceed two hours [12]. Primary PCI successfully achieves thrombolysis in myocardial infarction grade 3 flow in over 90% of patients, providing both immediate and sustained coronary reperfusion, outcomes that are significantly superior to those achieved with thrombolysis plus medical therapy. Although concomitant medical therapy is also required with PCI, it cannot replace the procedure.

The Danish Acute Myocardial Infarction 2 (DANAMI-2) trial demonstrated that, in patients treated at invasive centers within 120 minutes, primary PCI resulted in better outcomes than thrombolysis. Specifically, PCI was associated with a lower risk of reinfarction (13% vs. 18.5%; HR 0.66, 95% CI 0.49-0.89) and reduced mortality (26.7% vs. 33.3%; HR 0.78, 95% CI 0.63-0.97) compared with thrombolysis [13].

STEMI India, a national initiative aimed at improving STEMI management, promotes evidence-based reperfusion strategies to address disparities in care. It recommends primary PCI for patients with 24 × 7 cardiac catheterization laboratory access in urban centers and a PI approach, thrombolysis followed by PCI within three to 24 hours, for rural or delayed-access patients. This approach is supported by evidence from trials such as STREAM and STEP-PAMI.

The strategy is implemented through a hub-and-spoke model linking non-PCI centers to PCI-capable hubs, facilitating timely diagnosis, referral, and transfer. Successful pilots in Tamil Nadu, Gujarat, Goa, and Haryana have demonstrated reduced delays and improved outcomes. The Tamil Nadu project showed that a government-supported hub-and-spoke system, coordinated via telemedicine and social media, enhanced adherence to guideline-directed revascularization. The Goa project confirmed the feasibility of this approach but emphasized challenges such as the need for adequately trained staff and advanced technology to ensure sustainability [14-21].

Myth 3: Patients Must Be Completely Stabilized Before Transfer

Myth: Many Indian PCPs believe that patients should be hemodynamically stable and fully optimized before being transferred to a PCI-capable facility, which often leads to unnecessary delays in reperfusion.

Evidence-based reality: Evidence consistently shows that rapid transfer for primary PCI leads to better outcomes than prolonged stabilization attempts at non-PCI centers. Multiple studies demonstrate that transfer for primary PCI remains beneficial even with transport times of up to 90-120 minutes, provided delays are minimized [13,22]. Achieving these target times is critical, as each minute of delay in reperfusion increases myocardial injury and worsens outcomes [23].

Shortening the door-to-balloon (D2B) time has been repeatedly shown to provide a survival benefit, with improved outcomes even when D2B times are reduced from 90 minutes to under 60 minutes. In patients with STEMI complicated by cardiogenic shock, Scholz et al. [24] found that for those with first medical contact-to-device times of 60-180 minutes, every 10-minute delay was associated with 3.31 additional deaths per 100 PCI-treated patients, emphasizing the urgency of minimizing transfer and treatment delays in this high-risk group.

The concept of “stabilization” should therefore prioritize essential hemodynamic support during transfer rather than complete medical optimization before it. Patients requiring vasopressors, temporary pacing, or intra-aortic balloon support can be safely transported, as these interventions are typically managed more effectively at PCI-equipped centers [25].

Myth 4: The PI Strategy Is Too Complex for Primary Care

Myth: Indian PCPs often perceive the PI strategy as overly complex and requiring specialized expertise beyond the typical scope of primary care practice.

Evidence-based reality: The PI approach is simple to implement and does not require interventional cardiology skills. It involves administering standard fibrinolytic therapy followed by early transfer to a PCI-capable hospital for coronary angiography within three to 24 hours, with angioplasty performed if the culprit artery remains occluded or shows a critical lesion [26-28]. Early transfer is crucial, as many patients who appear clinically improved after thrombolysis may still have persistent or recurrent occlusion of the culprit vessel.

The STREAM trial demonstrated that the PI strategy can be effectively applied across diverse healthcare systems, offering outcomes comparable to primary PCI when timely transfer is ensured [18,29]. Supporting evidence from Indian studies, including the STEPP-AMI trial, confirmed that a PI approach using streptokinase, a widely available and affordable thrombolytic agent in India, was non-inferior to primary PCI, with similar 30-day and long-term clinical outcomes [17,30].

Myth 5: Primary PCI Is Perceived as Unaffordable for Most Indian Patients

Myth: Some Indian PCPs believe that the cost of primary PCI for STEMI management is prohibitively high for most patients.

Evidence-based reality: With the expansion of government-funded insurance schemes, primary PCI is now available free of cost in many hospitals across India. In addition, the government’s regulation and capping of stent prices have significantly reduced overall procedural costs in recent years. PCPs must recognize the favorable cost-benefit ratio of timely revascularization [31,32]. Early intervention prevents large infarcts and preserves myocardial function, thereby reducing the long-term risk of heart failure. This not only improves patient outcomes but also decreases future healthcare expenses associated with chronic heart failure management, such as costly medications and device therapy (Table 1) [32].

**Table 1:** Myths and truths regarding primary PCI and STEMI care PCI, percutaneous coronary intervention; PCP, primary care physician; PI, pharmacoinvasive; STEMI, ST-elevation myocardial infarction Sources: [5,7,9,11-24] Table credit: Kunal Mahajan

Myth	Truth
Primary PCI is dangerous or carries excessive risk.	Modern PCI is safe, with procedural risk significantly lower than the risk of untreated STEMI.
Thrombolysis is as effective as primary PCI for STEMI.	Primary PCI reduces mortality and adverse events significantly more than thrombolysis.
Admit and stabilize before shifting.	Early transfer to a PCI-capable hospital for primary PCI is optimal if it can be performed within the first two hours. If transfer within two hours is not possible, thrombolysis followed by immediate transfer should be performed. Ensuring safe and timely transport for primary or PI therapy improves outcomes.
STEMI ECG diagnosis and primary PCI/PI strategies are too complex for PCPs to understand.	Organized STEMI networks using standardized protocols and telemedicine (hub-and-spoke model) can facilitate quick ECG diagnosis, effective referral, and timely reperfusion in most patients.
Primary PCI is perceived as unaffordable for most Indian patients.	Public and private insurance schemes, along with government price regulation, have made PCI affordable in most healthcare settings.

Current state of STEMI care in India

Treatment Patterns and Outcomes

Data from major Indian registries present a concerning picture of STEMI management. The CREATE registry, encompassing 89 centers across India, revealed that fewer than 10% of patients received primary PCI [3]. The Kerala study reported similar findings, showing substantial variability in treatment approaches across hospitals [9].

Financial constraints continue to play a major role in treatment decisions. Several studies indicate that the availability of immediate financial resources or insurance coverage often determines whether a patient undergoes primary PCI or receives alternative treatments [33-36]. This economic reality contributes to significant disparities in access to optimal care, with patients from lower socioeconomic backgrounds being more likely to receive thrombolytic therapy.

Geographic and Infrastructure Challenges

India’s vast geography and uneven distribution of PCI-capable centers pose substantial challenges to timely STEMI management. Rural regions are particularly affected by limited emergency medical services, inadequate transportation infrastructure, and a shortage of specialists [1,16,17,37]. The hub-and-spoke model implemented in Tamil Nadu has sought to address these challenges, with encouraging but mixed outcomes [19,20].

Evidence Supporting Optimal STEMI Interventions

Primary PCI efficacy and safety: Strong evidence supports primary PCI as the most effective reperfusion strategy when performed within the recommended timeframes. Multiple randomized trials and meta-analyses have consistently demonstrated the superiority of primary PCI over thrombolysis [13,38,39]. Because the benefits of reperfusion are highly time dependent, minimizing treatment delays is critical; each 30-minute delay in reperfusion increases one-year mortality by approximately 7.5% [36].

PI strategy as an alternative: When primary PCI cannot be performed within the guideline-recommended window, a PI approach serves as a viable alternative. The STREAM trial demonstrated the non-inferiority of PI therapy compared with primary PCI in patients presenting within three hours of symptom onset. Indian studies evaluating PI strategies using streptokinase, the most widely available thrombolytic agent in the country, have reported similar efficacy and safety outcomes [18,27,40,41].

Discussion

Impact of Myths on Patient Care

The persistence of myths about primary angioplasty among Indian PCPs has significant implications for patient care. These misconceptions contribute to delayed referrals, inappropriate treatment choices, and suboptimal clinical outcomes. The unfounded fear of procedural risks, despite strong evidence supporting the excellent safety profile of primary PCI, often results in unnecessary delays that can worsen patient prognosis. Similarly, the belief that medical management alone is sufficient for STEMI reflects a fundamental misunderstanding of the pathophysiology of acute coronary occlusions. This misconception is particularly harmful, as it may lead to withholding lifesaving reperfusion therapy from patients who would otherwise benefit from timely intervention.

Barriers to Optimal Care Implementation

The barriers to effective STEMI management in India are multifactorial and occur at the system, physician, and patient levels. System-level barriers include inadequate healthcare infrastructure, limited availability of PCI-capable facilities, and poor coordination among healthcare centers. Physician-level barriers involve persistent knowledge gaps, fear of procedural complications, and communication challenges between PCPs and specialist centers [16,42,43].

Patient-level barriers include delayed recognition of symptoms, financial constraints, and sociocultural factors that influence healthcare-seeking behavior. The median time from symptom onset to hospital presentation in India is approximately 300 minutes, considerably longer than in developed countries, underscoring the urgent need for widespread public education initiatives (Table 2) [3,33,44].

**Table 2:** Barriers to STEMI care in India PCI, percutaneous coronary intervention; STEMI, ST-elevation myocardial infarction Sources: [3,14,29,38-40] Table credit: Kunal Mahajan

Category	Barrier
Awareness	Patient ignorance or underestimation of symptoms
Education	Low health literacy and limited awareness about heart attack symptoms
Primary care gaps	Inability of primary care providers to interpret ECGs or diagnose STEMI
Geographic	Remote and rural locations with limited access to PCI-capable centers
Logistical	Poor road conditions and a lack of organized ambulance services
Infrastructure	Lack of 24 × 7 primary PCI facilities in many hospitals
Transport	Inadequate prehospital systems and absence of ambulance-based ECG or triage
Financial	High cost of PCI and affordability issues in private or uninsured settings
Cultural myths	Fear that angioplasty (PCI) is dangerous or life-threatening
Training deficit	Lack of standardized STEMI protocols and training at peripheral or rural healthcare centers
Clinical	Silent or painless myocardial infarction presentations leading to delayed recognition
Referral delay	Poor coordination between peripheral and tertiary care centers

Economic Considerations

Cost-effectiveness analyses consistently demonstrate that primary PCI offers superior value compared with thrombolytic therapy, providing lower lifetime healthcare costs and improved quality-adjusted life years [32]. The PI strategy also presents significant economic advantages in resource-limited settings, combining the immediate accessibility of fibrinolysis with the enhanced clinical outcomes of mechanical reperfusion [45,46].

Recommendations for Improvement

Education and training initiatives: Comprehensive educational programs for Indian PCPs should directly address the prevalent myths surrounding STEMI management and provide practical, evidence-based guidance on diagnosis and treatment. These initiatives must emphasize the safety and efficacy of primary PCI, the role of PI strategies, and the importance of early recognition and prompt referral [1,47].

System-level interventions: Healthcare systems should implement standardized protocols for STEMI management that clearly outline the roles and responsibilities of PCPs, emergency physicians, and interventional cardiologists. These protocols should include stepwise decision algorithms, time-bound targets, and quality metrics to ensure consistency and accountability across diverse healthcare settings [14,48,49].

Technology and communication enhancement: Modern technology offers powerful tools to improve coordination and reduce delays in STEMI care. Telemedicine platforms can facilitate remote consultations and ECG interpretations, particularly beneficial in rural and underserved regions [17]. Pre-hospital ECG transmission systems and direct catheterization laboratory activation protocols have been shown to significantly shorten treatment times [19,20].

Limitations and Future Directions

AI is gradually transforming the STEMI care continuum, from initial patient presentation to the rapid activation of the catheterization laboratory. AI-powered ECG interpretation tools and mobile applications are helping clinicians identify STEMI earlier and more accurately. These algorithms can detect subtle ECG changes that might be overlooked by even experienced physicians, providing real-time alerts that accelerate diagnosis and treatment decisions.

By connecting peripheral centers, ambulances, and PCI-capable hospitals, AI-driven systems enable more efficient coordination and reduce critical delays. In resource-limited settings, decision support tools empower clinicians to make timely, evidence-based choices, bridging the gap between urban and rural cardiac care. However, these technologies remain in development and rely on high-quality data, rigorous validation, and careful oversight to ensure accuracy, reliability, and ethical use. As AI continues to evolve, its potential is clear: to make STEMI care faster, smarter, and more equitable, ensuring that every patient, regardless of location, has access to lifesaving interventions.

## Conclusions

STEMI management in India continues to face major challenges due to persistent myths and misconceptions among PCPs, which contribute to treatment delays and the underutilization of evidence-based reperfusion strategies. Despite robust evidence confirming that primary PCI is safe, effective, and superior to thrombolytic therapy, many patients still do not receive timely interventions. The PI strategy provides an important alternative when immediate PCI is not feasible, particularly in resource-limited or geographically remote settings. Addressing these barriers requires a multifaceted approach encompassing targeted physician education, widespread implementation of standardized care protocols, the development of hub-and-spoke referral networks, and the integration of technologies such as telemedicine and pre-hospital ECG systems. Equally crucial is the adaptation of international STEMI guidelines to India-specific contexts that account for local disparities in infrastructure, healthcare workforce, and socioeconomic conditions. Through such coordinated and context-sensitive efforts, India can ensure equitable access to timely reperfusion therapy and achieve substantial improvements in STEMI outcomes nationwide.
